# Caution in interpreting disease‐modification claims with lecanemab: Selective reporting and causal inference

**DOI:** 10.1002/alz.71486

**Published:** 2026-05-21

**Authors:** Lon S. Schneider, Richard E. Kennedy, Gary Cutter

**Affiliations:** ^1^ Keck School of Medicine University of Southern California Los Angeles California USA; ^2^ University of Alabama Birmingham Birmingham Alabama USA

1

To the Editor,

We write to express concerns regarding the report by van Dyck and colleagues describing long‐term outcomes from the open‐label extension (OLE) of the Phase 3 *Clarity AD* trial.^1^ The report presents methodological and interpretive problems about clinical effectiveness that undermine its scientific reliability and risks misleading readers.

The OLE is a non‐randomized extension in which participants receive active treatment, and the authors acknowledge that no formal hypothesis testing was performed. Yet the report repeatedly adopts confirmatory language and advances causal and disease‐modifying interpretations that the design cannot support. Claims of continued accrual of benefit, lack of convergence or “catch‐up” among delayed‐start participants, and disease‐modifying effects are not substantiated by the scant data, figures, or analyses provided.

Assertions of long‐term efficacy rely almost entirely on graphical depictions of trajectories or slopes, without disclosure of numerical values, effect sizes, confidence intervals, or tabulated outcomes. Readers are presented with figures labeled with phrases such as “continued separation,” “sustained slowing,” or “expanding benefit,” yet they are denied the quantitative data required to assess effect magnitude, variability, clinical relevance, or robustness. This substitution of visual suggestion for transparent quantitative reporting impedes independent evaluation and falls short of accepted standards for clinical trial reporting.^2^

The delayed‐start interpretation is likewise inadequately supported, resting mainly on visual separation and visit‐level contrasts rather than on a prespecified post‐switch longitudinal estimand.^3^ Although the Statistical Analysis Plan (SAP) specifies longitudinal modeling of the Clinical Dementia Rating–Sum of Boxes (CDR‐SB) across the randomized and OLE periods, the report does not present the post‐switch slope‐based analyses needed to test delayed‐start hypotheses once both groups receive active lecanemab. A properly specified delayed‐start analysis (e.g., a piecewise longitudinal model with a breakpoint at Month 18 and estimands that address intercurrent events and dropout) is required to determine whether delayed‐start participants converge toward early‐start participants or progress in parallel. Absent such analyses, inferences of disease modification are not supported by the presented evidence.

The report further relies on comparisons with an external matched cohort from the Alzheimer's Disease Neuroimaging Initiative (ADNI) to claim long‐term efficacy. Although a supplement describes the matching, analytic details needed for appraisal are not provided. Statistical details underlying these comparisons are largely absent, including key elements of the analysis, covariates, missing‐data assumptions, and sensitivity analyses to address the large discrepancy in dropouts (35% in early‐start vs 60% in ADNI at Month 36). Although limitations of historical controls are acknowledged, the comparisons function rhetorically to bolster claims of durable benefit.

Results from post hoc subgroup analyses defined by amyloid positron emission tomography (PET) <60 Centiloid (CL) or “low” tau PET thresholds are highlighted and used to support arguments for early treatment initiation. These analyses were not pre‐specified, relied on multiple threshold definitions, and are vulnerable to multiplicity, regression to the mean, and selection bias, as randomization no longer guarantees balance. Despite these limitations, the report emphasizes favorable findings in these subgroups without sufficient statistical detail or a clear distinction between exploratory analyses and inferential conclusions. Corresponding analyses of amyloid PET ≥60 CL or high tau PET subgroups are absent, and the report offers no evidentiary basis—beyond assertion—for the novel claim: “the first time lower baseline amyloid levels have been linked to better treatment outcomes.”

Safety reporting, although more extensive than efficacy reporting, also raises concerns regarding transparency. Exposure‐adjusted rates of amyloid‐related imaging abnormalities (ARIA) are provided; however, cumulative incidence—particularly of ARIA involving hemorrhage (ARIA‐H) with longer duration of exposure—is not clearly presented or contextualized. Conclusions that ARIA is not associated with clinical progression are supported primarily by reference to “Data on File,” rather than by results available for review.

In the Discussion, causal language exceeds what can be supported by a descriptive OLE. Phrases such as “benefit continued to accrue over three years,” “clear demonstration of disease‐modifying effects,” “strong support for early and continuous treatment,” and assertions that delayed‐start participants “do not catch up,” imply irreversible disease modification despite the acknowledged design limitations. Exploratory, non‐randomized observations are framed as confirmatory evidence.

Addressing these issues requires more than editorial qualification. At minimum, the authors should: (1) provide complete numerical tabulation of all long‐term efficacy outcomes shown graphically; (2) fully report statistical methods and results for OLE and ADNI cohort comparisons, including approaches to informative censoring; (3) disclose analyses conducted according to the SAP; and (4) make underlying data available for independent evaluation, consistent with current standards for trial transparency.

Absent these corrections, the manuscript overstates what can be reasonably inferred from an OLE and conveys unwarranted confidence in claims of long‐term efficacy and disease modification. Whether lecanemab is disease modifying will be determined in ongoing randomized controlled trials with extended treatment duration such as AHEAD 3‐45. At present, that standard has not been met.

## CONFLICT OF INTEREST STATEMENT

L.S.S. reports grants from National Institutes of Health (NIH) R01 AG054434 (Delivery of Essential Fatty Acids to the Brain), P30 AG066530 USC Alzheimer Disease Research Center (USC ADRC), R01 AG062687, R01 AG051346, R01 AG055444, P01 AG02350, R01 AG053267 Dominantly Inherited Alzheimer Network‐Treatment Unit (DIAN‐TU), R01 AG074983 (Aβ and tau vaccines), R01 AG063826, State of California California Alzheimer Disease Center (CADC) 15‐10291; grants and personal fees from Eli Lilly/Avid, grants and personal fees from Roche/Genentech; grants/contracts from Eisai, Biogen, and Bristol Myers Squibb (BMS); grants from Washington University, St. Louis/NIA DIAN‐TU; grants from UC San Diego Alzheimer Disease Cooperative Study (ADCS); personal fees from Neurim, Ltd, Immunobrain Checkpoint, Ltd, BioVie, Lexeo, Lighthouse, AC Immune, Actinogen, Pharmatrophix, Linus Health, Lundbeck, Novo Nordisk, Muna Ltd, Ono Ltd, and Vivli.org, outside the submitted work, and from The Della Martin Foundation endowment within the past 36 months.

R.E.K. reports grants from NIH R01AG096921 (Impact of Undiagnosed Dementia on Healthcare Utilization Across Geographic Regions), R01AG081433 (Role of GlcSph in cognitive deficits in Lewy body dementias), R21AG084218 (Comprehensive Assessment of Delirium Risk due to Medications), T32NS095775 (UAB Training Program in Neurodegeneration), R01ES034846 (The role of lysosomal impairment in trichloroethylene induced Parkinsonian neurodegeneration), R61AG088966 (Equity in Access to Alzheimer's Disease Pharmacologic Treatment for African American and White Patients in Alabama: A Mixed Methods Study to Inform Recommendations (EquAAL)), Administration for Community Living 90DPTB0015 (UAB Traumatic Brain Injury Model Systems), Veterans Health Administration SDR 23‐115‐2 (A Hybrid Effectiveness‐Implementation Study of the Pride in All Who Served Program for LGBTQ+ Veterans (AFFIRM Study)), Veterans Health Administration IIR 23‐027‐3 (Preventing Loss of Independence through Exercise in Community Living Centers: An Effectiveness‐Implementation Trial), Michael J. Fox Foundation for Parkinson's Research ASAP‐025191 (Understanding and Manipulating Cellular and Circuit‐level Vulnerability to Neurodegeneration in Parkinson's Disease).  He also serves as Data Safety Monitoring Board (DSMB) Chair for NIH grant R61AG065619 (Enhancing Sleep Quality for Nursing Home Residents with Dementia: Pragmatic Trial of an Evidence‐Based Frontline Huddling Program).

G.C. participated in Data and Safety Monitoring Boards for the following companies: Applied Therapeutics, AI Therapeutics, AMO Pharma, Astra‐Zeneca, Avexis Pharmaceuticals, Bristol Meyers Squibb/Celgene, CSL Behring, Cynata Therapeutics, Horizon Pharmaceuticals, Immunic, Karuna Therapeutics, Kezar Life Sciences, Mapi Pharmaceuticals Ltd, Merck, Mitsubishi Tanabe Pharma Holdings, Opko Biologics, Prothena Biosciences, Novartis, Pipeline Therapeutics, Regeneron, Sanofi‐Aventis, Reata Pharmaceuticals, Teva Pharmaceuticals, United BioSource LLC, University of Texas Southwestern, University of Pennsylvania, and Visioneering Technologies, Inc. G.C. participated in consulting or advisory boards of the following companies: Alexion, Antisense Therapeutics, Avotres, Biogen, Clene Nanomedicine, Clinical Trial Solutions LLC, Endra Life Sciences, Cognito Therapeutics, Entelexo Biotherapeutics, Inc., Genzyme, Genentech, GW Pharmaceuticals, Hoya Corporation, Immunic, Immunosis Pty Ltd, Klein‐Buendel Incorporated, Linical, Merck/Serono, Novartis, Perception Neurosciences, Protalix Biotherapeutics, Regeneron, Roche, SAB Biotherapeutics, and Sapience Therapeutics; and is employed by the University of Alabama at Birmingham and is the president of Pythagoras, Inc., a private consulting company located in Birmingham, AL, USA. Authors disclosure are available at Supporting Information.

## Supporting information




Supporting Information
