# Supplementary material for: Caution in interpreting disease‐modification claims with lecanemab: Selective reporting and causal inference
Source: Alzheimers Dement. 2026 May 21;22(5):e71486. doi: 10.1002/alz.71486 (PMC13239976; doi:10.1002/alz.71486)
Supplement: Supplementary file 1 — Supporting Information [file ALZ-22-e71486-s001.pdf]

ICMJE DISCLOSURE FORM

Date: 2/14/2026

Your Name: Lon S. Schneider

Manuscript Title: *Letter to Editor*

Manuscript Number (if known): ADJ-D-26-00280

In the interest of transparency, we ask you to disclose all relationships/activities/interests listed below that are related to the content of your manuscript. "Related" means any relation with for-profit or not-for-profit third parties whose interests may be affected by the content of the manuscript. Disclosure represents a commitment to transparency and does not necessarily indicate a bias. If you are in doubt about whether to list a relationship/activity/interest, it is preferable that you do so.

The author's relationships/activities/interests should be defined broadly. For example, if your manuscript pertains to the epidemiology of hypertension, you should declare all relationships with manufacturers of antihypertensive medication, even if that medication is not mentioned in the manuscript.

In item #1 below, report all support for the work reported in this manuscript without time limit. For all other items, the time frame for disclosure is the past 36 months.

		**Name all entities with whom you have this relationship or indicate none (add rows as needed)**	**Specifications/Comments (e.g., if payments were made to you or to your institution)**
		Time frame: Since the initial planning of the work	
1	All support for the present manuscript (e.g., funding, provision of study materials, medical writing, article processing charges, etc.) **No time limit for this item.**	☐ None	
		Della Martin Foundation	Endowment
		NIH P30 AG066530	USC ADRC
		Time frame: past 36 months	
2	Grants or contracts from any entity (if not indicated in item #1 above).	☐ **None**	
		NIH R01 AG062687	NIH R01 AG051346
		NIH R01 AG055444	NIH P01 AG02350
		NIH R01 AG053267	NIH R01 AG074983
		NIH R01 AG063826	Biohaven
		Biogen	Eisai
		Eli Lilly	Banner Alzheimer's Institute
		F. Hoffmann-La Roche Ltd/ Genentech, Inc	Voyager

		Name all entities with whom you have this relationship or indicate none (add rows as needed)	Specifications/Comments (e.g., if payments were made to you or to your institution)
**3**	Royalties or licenses	☒ **None**	
**4**	Consulting fees	☐ **None**	
		AC Immune	Cortexyme
		Athira	BioVie
		ImmunoBrain, Ltd	Actinogen
		Lexeo	Lundbeck
		Lighthouse	Linus Health
		Merck	Novo-Nordisk
		Neurim Ltd	Muna Ltd
		Ono Ltd	Pharmatrophix
		Vivli.org	Bristol Myers Squibb
**5**	Payment or honoraria for lectures, presentations, speakers bureaus, manuscript writing or educational events	☒ **None**	
**6**	Payment for expert testimony	☒ **None**	
**7**	Support for attending meetings and/or travel	☐ **None**	
		Della Martin Foundation	
		USC ATRI/ACTC	
		UCSD ADCS	
**8**	Patents planned, issued or pending	☒ **None**	
**9**	Participation on a Data Safety	☐ **None**	

		Name all entities with whom you have this relationship or indicate none (add rows as needed)	Specifications/Comments (e.g., if payments were made to you or to your institution)
	Monitoring Board or Advisory Board	Merck	BMS
		Genentech	Voyager
**10**	Leadership or fiduciary role in other board, society, committee or advocacy group, paid or unpaid	☒ **None**	
**11**	Stock or stock options	☒ **None**	
**12**	Receipt of equipment, materials, drugs, medical writing, gifts or other services	☒ **None**	
**13**	Other financial or non-financial interests	☒ **None**	

Please place an "X" next to the following statement to indicate your agreement:

☒ I certify that I have answered every question and have not altered the wording of any of the questions on this form.

ICMJE DISCLOSURE FORM

Date: 2/15/2026

Your Name: Richard E Kennedy

Manuscript Title: Letter to Editor

Manuscript Number (if known): ADJ-D-26-00280

In the interest of transparency, we ask you to disclose all relationships/activities/interests listed below that are related to the content of your manuscript. "Related" means any relation with for-profit or not-for-profit third parties whose interests may be affected by the content of the manuscript. Disclosure represents a commitment to transparency and does not necessarily indicate a bias. If you are in doubt about whether to list a relationship/activity/interest, it is preferable that you do so.

The author's relationships/activities/interests should be defined broadly. For example, if your manuscript pertains to the epidemiology of hypertension, you should declare all relationships with manufacturers of antihypertensive medication, even if that medication is not mentioned in the manuscript.

In item #1 below, report all support for the work reported in this manuscript without time limit. For all other items, the time frame for disclosure is the past 36 months.

		Name all entities with whom you have this relationship or indicate none (add rows as needed)	Specifications/Comments (e.g., if payments were made to you or to your institution)
**Time frame: Since the initial planning of the work**			
1	All support for the present manuscript (e.g., funding, provision of study materials, medical writing, article processing charges, etc.) **No time limit for this item.**	☐ None	
		NIH/NIA Grant R01AG096921 (R. Kennedy, M. Crowe A. Lo, MPI	
		NIH/NIA Grant P30AG086401 (E. Roberson, PI)	
			Click the tab key to add additional rows.
**Time frame: past 36 months**			
**2**	Grants or contracts from any entity (if not indicated in item #1 above).	☐ **None**	
		Multiple grants from NIH, VHA, Administration for Community Living, and Michael J. Fox Foundation unrelated to the current work	
**3**	Royalties or licenses	☒ **None**	

		Name all entities with whom you have this relationship or indicate none (add rows as needed)	Specifications/Comments (e.g., if payments were made to you or to your institution)
**4**	Consulting fees	☒ **None**	
**5**	Payment or honoraria for lectures, presentations, speakers bureaus, manuscript writing or educational events	☒ **None**	
**6**	Payment for expert testimony	☒ **None**	
**7**	Support for attending meetings and/or travel	☒ **None**	
**8**	Patents planned, issued or pending	☒ **None**	
**9**	Participation on a Data Safety Monitoring Board or Advisory Board	☐ **None**	
		NIH/NIA Grant R61AG065619; L. Snow, PI	
**10**	Leadership or fiduciary role in other board, society, committee or advocacy group, paid or unpaid	☒ **None**	

		Name all entities with whom you have this relationship or indicate none (add rows as needed)	Specifications/Comments (e.g., if payments were made to you or to your institution)
**11**	Stock or stock options	☒ **None**	
**12**	Receipt of equipment, materials, drugs, medical writing, gifts or other services	☒ **None**	
**13**	Other financial or non-financial interests	☒ **None**	

Please place an "X" next to the following statement to indicate your agreement:

☒ I certify that I have answered every question and have not altered the wording of any of the questions on this form.

ICMJE DISCLOSURE FORM

**Date:**	2/14/2026
**Your Name:**	Gary Cutter
**Manuscript Title:**	Letter to editor, lecanemab
**Manuscript Number (if known):**	ADJ-D-26-00280

In the interest of transparency, we ask you to disclose all relationships/activities/interests listed below that are related to the content of your manuscript. "Related" means any relation with for-profit or not-for-profit third parties whose interests may be affected by the content of the manuscript. Disclosure represents a commitment to transparency and does not necessarily indicate a bias. If you are in doubt about whether to list a relationship/activity/interest, it is preferable that you do so.

The author's relationships/activities/interests should be defined broadly. For example, if your manuscript pertains to the epidemiology of hypertension, you should declare all relationships with manufacturers of antihypertensive medication, even if that medication is not mentioned in the manuscript.

In item #1 below, report all support for the work reported in this manuscript without time limit. For all other items, the time frame for disclosure is the past 36 months.

		**Name all entities with whom you have this relationship or indicate none (add rows as needed)**	**Specifications/Comments (e.g., if payments were made to you or to your institution)**
**Time frame: Since the initial planning of the work**			
1	All support for the present manuscript (e.g., funding, provision of study materials, medical writing, article processing charges, etc.) **No time limit for this item.**	☒ None	
			Click the tab key to add additional rows.
**Time frame: past 36 months**			
**2**	Grants or contracts from any entity (if not indicated in item #1 above).	☐ **None**	
		NIH Project Number: R01AG070049	Institution
		Project Number: U54 NS115054	UAB for all
		Project Number: 2U01AR071133	
		Project Number: R01 AI148359	
		Project Number: R01 HL120338	
		Project Number: U24 HL155807	
		Project Number: 90IFRE0073	
		Project Number: U54 DK137307	
		Project Number: 5U2CDK133422-02	
		Project Number: U24 DK137318	
		Project Number: R01 HD112994	
		PCORI Outcomes of Fragility Fracture	

		Name all entities with whom you have this relationship or indicate none (add rows as needed)	Specifications/Comments (e.g., if payments were made to you or to your institution)
		NMSS dynamics of vascular changes during exercise on MS patients with hypertension	
		Project Number: R01DK078244	
		Project Number: 1P30AG086401-01	
		Project Number: R01 AR073004	
**3**	Royalties or licenses	☒ **None**	
**4**	Consulting fees	☐ **None**	
		Alexion, Antisense Therapeutics/Percheron, Avotres, Biogen, Clene Nanomedicine, Clinical Trial Solutions LLC, Endra Life Sciences, Cognito Therapeutics, Genzyme, Genentech, Hoya Corporation, Immunic, Immunosis Pty Ltd, Klein-Buendel Incorporated, Kyverna Therapeutics, Inc. , Linical, Merck/Serono, Noema, Neurogenesis, Perception Neurosciences, Protalix Biotherapeutics, Regeneron, Revelstone Consulting, Roche, SAB Biotherapeutics, Sapience Therapeutics, Scott&Scott LLP, Tenmile.	Payments to me
**5**	Payment or honoraria for lectures, presentations, speakers bureaus, manuscript writing or educational events	☒ **None**	
**6**	Payment for expert testimony	☒ **None**	

		Name all entities with whom you have this relationship or indicate none (add rows as needed)	Specifications/Comments (e.g., if payments were made to you or to your institution)
**7**	Support for attending meetings and/or travel	☒ **None**	
**8**	Patents planned, issued or pending	☒ **None**	
**9**	Participation on a Data Safety Monitoring Board or Advisory Board	☐ **None**	
		Applied Therapeutics, AI therapeutics, AMO Pharma, Argenx, Astra-Zeneca, Avexis Pharmaceuticals, Bristol Meyers Squibb, CSL Behring, Cynata Therapeutics, DiamedicaTherapeutics, Horizon Pharmaceuticals, Immunic, Inhibrix, Karuna Therapeutics, Kezar Life Sciences, Medtronic, Merck, Meiji Seika Pharma, Mitsubishi Tanabe Pharma Holdings, Prothena Biosciences, Novartis, Pipeline Therapeutics (Contineum), Regeneron, Sanofi-Aventis, Teva Pharmaceuticals, United BioSource LLC, University of Texas Southwestern.	Payments to me
**10**	Leadership or fiduciary role in other board, society, committee or advocacy group, paid or unpaid	☐ **None**	
		Birmingham Jewish Federation	None
		Birmingham Jewish Foundation	None
		Graffman Endowment Committee	None
**11**	Stock or stock options	☒ **None**	
**12**	Receipt of equipment, materials, drugs, medical writing, gifts or other services	☒ **None**	

		Name all entities with whom you have this relationship or indicate none (add rows as needed)	Specifications/Comments (e.g., if payments were made to you or to your institution)
**13**	Other financial or non-financial interests	☒ **None**	

Please place an "X" next to the following statement to indicate your agreement:

☒ I certify that I have answered every question and have not altered the wording of any of the questions on this form.
